# Intensive care for the adult population in Ireland: a multicentre study of intensive care population demographics

**DOI:** 10.1186/cc7018

**Published:** 2008-09-18

**Authors:** The Irish Critical Care Trials Group

**Affiliations:** 1 22 Merrion Square North, Dublin 2, Ireland

## Abstract

**Introduction:**

This prospective observational study was conducted to describe the nature of the intensive care population across Ireland, identify adherence to international benchmarks of practice, and describe patient outcomes in critically ill patients.

**Methods:**

A prospective observational multicentre study of demographics and organ failure incidence was carried out over a 10-week period in 2006 across the intensive care units (ICUs) of 14 hospitals in both the Republic and Northern Ireland.

**Results:**

In total, there were 1,029 patient episodes entered across 14 ICUs. Emergency admissions accounted for 70% of episodes. Admissions after major elective surgery accounted for 20.5% of admissions. The mean length of ICU stay was 5.7 days, with a median of 2 days. Severe sepsis was identified in 35% of patients during their ICU admission. Mechanical ventilation was used in 70.7% of all patients admitted, of whom 26.9% had acute lung injury. Acute kidney injury occurred in 28% of all patients. Interhospital transfers were undertaken in 85 (8.3%) patients. The overall intensive care mortality of the study population was 19%.

**Conclusions:**

Intensive care medicine in Ireland serves a patient population with high requirement for mechanical ventilation and support of the function of multiple organs. The overall mortality compares favourably with international benchmarks.

## Introduction

The Irish Critical Care Trials Group (ICCTG) was formed in 2006 with the aim of improving the capacity to conduct high-quality clinical research in the critically ill in Ireland. For many years in Ireland, clinicians in critical care medicine have participated in international multicentre trials and collaborated with such trials groups such as the European Society of Intensive Care Medicine and more recently its European Critical Care Research Network Group, and the Australia and New Zealand Intensive Care Clinical Society Trials Group, or have conducted focused studies within their own critical care population.

In order to inform hypotheses, feasibility and design of multicentre clinical trials, there was a need to first define the epidemiology of the potential study population. The ability of the participating units to complete the study was an important outcome measure for further collaborative ICCTG work. Accordingly, the ICCTG conducted a national audit of adult patient demographics and organ failure incidence in intensive care. The ICCTG has decided to address paediatric intensive care and high dependency as a separate study.

## Materials and methods

A prospective 10-week (August to October 2006) national audit of patient demographics and organ failure incidence in intensive care in Ireland was conducted in consecutive patient admissions across the 14 general intensive care units (ICUs) that form the ICCTG. All of the nine Irish University teaching hospital ICUs participated. All participating ICUs would be defined [1] as ICS level 3, supported by centralization of national specialties (for example, neurosurgery and cardiothoracic surgery). All three neurosurgical ICUs for Ireland were included in the study. These hospitals have available to them a total of 97 ICU beds in the Republic of Ireland, and 37 ICU beds in Northern Ireland, representing approximately 50% and 68% of ICU beds in those regions, respectively. Research ethics committee or audit committee approval was obtained as per local hospital or jurisdiction policy pertaining to audit, with need for informed consent waived.

Standard demographic data, including individual organ and total Sequential Organ Failure Assessment (SOFA) [2] score, were recorded daily each morning between 08:00 hours and 10:00 hours in all patients until ICU discharge. The SOFA score is composed of scores from six organ systems, graded from 0 to 4 according to the degree of dysfunction/failure. Organ systems considered in the SOFA are as follows: respiratory (arterial oxygen tension [PaO_2_]/fraction of inspired oxygen [FiO_2_]), cardiovascular (blood pressure, vasoactive drugs), renal (creatinine and diuresis), haematological (platelet count), neurological (Glasgow Coma Scale score) and liver (bilirubin).

Standard accepted international criteria were used to define sepsis syndrome [3], with the data entry requiring confirmation of each organ dysfunction as per the criteria. Site of sepsis was based on physician diagnosis.

Acute lung injury (ALI)/acute respiratory distress syndrome (ARDS) was defined using the American European Consensus Conference [4] criteria for ALI/ARDS, including the following: acute onset of bilateral chest radiographic infiltrates; PaO_2_/FiO_2 _ratio below 40 kPa for ALI and under 27 kPa for ARDS; and absence of cardiac failure or left atrial hypertension (assessed clinically, echocardiographically, or with invasive monitoring) and need for invasive ventilation.

The RIFLE (Risk, Injury, Failure, Loss of kidney function, End-stage kidney disease) criteria [5], as proposed by the Acute Dialysis Quality Initiative group, were used to describe the spectrum of acute kidney injury (AKI) from 'at risk' to 'established' renal failure.

Severe brain injury was defined according to aetiology of traumatic, spontaneous subarachnoid haemorrhage, stroke, meningitis, or encephalitis, and associated Glasgow Coma Scale score on admission to intensive care.

A form summarizing all ICU admissions and discharges during the previous 24 hours was submitted daily. The total ICU admissions determined from the daily report was used to confirm that all patients were included. In addition, this served as a control function to ensure that the participating centres remained active and screened patients throughout the study period. All data were collected using standard definitions as above, using a standardised electronic spreadsheet developed with the Clinical Research Support Centre (CRSC), in which tick boxes are used to record each defined variable within predefined ranges. An ALI entry required the patient to meet the consensus conference criteria described above, with a 'yes'/'no' tick box entry. The PaO_2_/FiO_2 _ratio defined progression from ALI to ARDS. The principal ICU investigator at each centre was responsible for data validation before submission to the coordinating CRSC. Telephone and e-mail assistance from the CRSC was available. The data were uploaded by batch data entry into the study database at the CRSC and then reviewed for inconsistencies and data entry errors. Any inconsistencies were then resolved by communication with reporting sites.

### Statistical analysis

Proportions were used as descriptive statistics for categorical variables, mean (standard deviation) for normally distributed continuous variables, and median (interquartile range) for non-normally distributed continuous variables.

## Results

**Figure 1:**
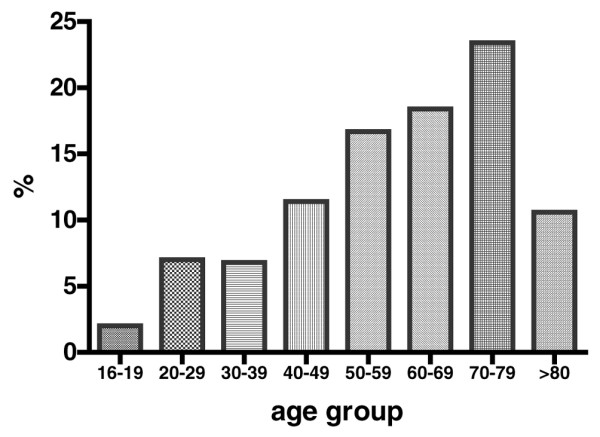
Age profile of admissions.

A total of 1,029 patient episodes with completed datasets were identified over the 10-week study period. Data were not available for those centres unable to adhere to the data collection requirements. The patients' epidemiological characteristics are described in Table 1. Emergencies comprised 723 (70%) of all admissions and interhospital transfers 85 (8.3%), yielding a nonelective admission total of 808 (78.5%) patients. The mean age of patients in the study was 57 (standard deviation 20.8) years; 62% were male. The age profile is illustrated in Figure 1. The mean length of ICU stay for the study was 6.3 days and the median 2 days (interquartile range 1 to 7 days), not censored for mortality.

**Table 1:** Epidemiological characteristics of the patients

Characteristic	Value
Patient episodes (n)	1,029
Age (years; mean ± SD)	57 ± 20.8
Sex (male; n [%])	626 (62%)
Admission type (n [%])	
Scheduled surgery	211 (20%)
Emergency admissions	808 (78.5%)
SOFA (mean ± SD)	
1st day: all	5.45 ± 3.8
1^st ^day: survivors^a^	5.3 ± 3.4
1^st ^day: nonsurvivors^b^	8.9 ± 3.7
ICU length of stay (median [IQR])	2 (1 to 7)
ICU mortality (n [%])^c^	162 (17.6%)
Readmissions (n [%])	72 (7.5%)
Readmission mortality (n [%])	16 (23%)

^a^Mean ± SD SOFA-max 6.5 ± 3.7. ^b^Mean SOFA-max 11.3 ± 3.9. ^c^Unadjusted for case mix, excluding readmissions. ICU outcome data available for 922 first time admissions and 69 readmissions. ICU, intensive care unit; SD, standard deviation; SOFA, Sequential Organ Failure Assessment

The mean SOFA score on admission for intensive care survivors was 5.3, and for nonsurvivors it was 8.9, with the degree of organ dysfunction on admission correlating with mortality (*P *< 0.001). Single organ failure was noted in 26% of admissions, the commonest such failure being respiratory. More than one organ failure was present in 62% of admissions (Table 2).

**Table 2:** Characteristics of organ failure, sepsis, lung injury and acute kidney injury

	Number (% of total study population)	Mortality (% within subgroup)
Organ failures on admission		
0^a^	111 (10.8)	1 (1)
1	273 (26.6)	24 (8.8)
2	258 (25.1)	32 (12.4)
3	167 (16.3)	36 (21.6)
4	123 (12)	38 (30)
5	58 (5.6)	25 (43)
6	35 (3.4)	21 (60)
Severe sepsis		
On admission	235 (22.8)	
Day 1	51 (4.9)	
Day 2 onwards	80 (7.8)	
Mortality sepsis group		90 (24.6)
Mechanical ventilation	728 (70.7)	
ALI/ARDS	196 (19)	63 (32.3)
AKI	289 (28)	
In sepsis	186 (51)	
In ALI/ARDS	84 (43)	
RIFLE 'Failure' category	132 (13)	50 (38)

^a^Major elective surgery comprised 20% of ICU admissions. AKI, acute kidney injury; ALI, acute lung injury; ARDS, acute respiratory distress syndrome; RIFLE, Risk, Injury, Failure, Loss of kidney function, End-stage kidney disease.

The commonest reason for ICU admission was severe sepsis, accounting for 235 (22.8%) of admissions. A further 131 (12.7%) patients had an episode of sepsis identified during their ICU admission, 51 patients at less than 2 days, and 80 patients at greater than 2 days. In total 366 (35%) of all patients had an episode of severe sepsis during their ICU admission. Respiratory sepsis was the most frequent site of sepsis, accounting for 10.3% of all admissions. Abdominal sepsis comprised 8% of admissions. Sepsis with associated organ dysfunction was present in 86% of patients categorized as having sepsis on admission, with 80% having cardiovascular dysfunction. Two organ failures were present in 79.8% of patients with an episode of severe sepsis. Steroid therapy in sepsis was common (46.7%), the majority of whom (93%) had cardiovascular organ dysfunction at the time. Recombinant activated protein C was administered to 21 patients with severe sepsis.

**Figure 2:**
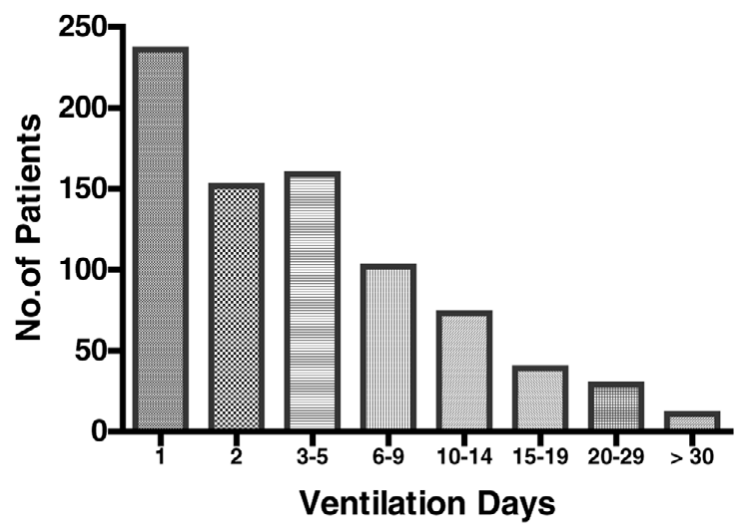
Duration of mechanical ventilation.

Invasive mechanical ventilation (via tracheal intubation) was received by 728 (70.7%) of patients. The duration of mechanical ventilation ranged from 1 to 48 days, with a median of 3 days and mean of 5.5 days (Figure 2). Of the 728 patients who required mechanical ventilation, 196 (26.9%) fulfilled ALI criteria, as reported previously [6].

Noninvasive ventilation (NIV) was utilized in 137 patients overall, 69 patients (6.7%) on admission and 68 at a later stage in their ICU stay. Of the 69 patients with NIV on admission, a complete dataset was available for 66. Two patients required only a single day in ICU, did not require invasive ventilation and were discharged. A further 19 progressed to invasive ventilation (29%). The study was not designed to clarify reasons for choice of NIV at admission, or the reasons for use of NIV after extubation (for example, elective therapy versus extubation failure).

AKI defined by RIFLE criteria was present in 289 (28%) patients at some point during the admission. Of the 147 patients satisfying Risk or Injury criteria initially, 34.7% progressed to a more severe level of injury while in the ICU. The incidence of AKI in elective surgery patients was 8.5%, versus 44% in emergency admissions overall and 27% in interhospital transfers. The incidence of AKI in patients with ARDS was 43%. The incidence of AKI was 51% in patients with a diagnosis of sepsis on admission (13% Risk, 10% Injury and 28% Failure). The length of ICU stay increased as kidney injury increased (4.8, 7.8, 8.1 and 12.1 days for no AKI, Risk, Injury and Failure, respectively). Of the 1,029 admissions, in 69 (6.7%) renal replacement therapy (RRT) was instituted.

Severe brain injury was reported in 128 patients, of whom 72 (56%) had suffered a traumatic brain injury, 31 (24%) spontaneous subarachnoid haemorrhage, 16 (12.4%) cerebral infarct, seven (5.4%) intracranial haemorrhage, three (2.3%) meningitis and six (4.7%) injury not specified. In total, 37 patients were cared for in a non-neurosurgical centre of the participating centres, and 91 in a neurosurgical centre. Of those 91, there were 32 transfers to the neurosurgical centres, of which nine came from participating units. Of the 91 admissions to neurosurgical centres, 23 had suffered a traumatic brain injury.

The time of discharge from the ICU was sought in order to define pressure of new admissions to the units out of hours. A total of 197 (19.1%) of all discharges occurred between the hours of 18:00 and 08:00.

For patients discharged and not readmitted later to the ICU, the crude mortality rate (not adjusted for case mix) was 17.6%. Of alive discharges from ICU, the readmission rate to the ICUs was 7.5%, with a mortality of 23%. Analysis of the major disease categories in the audit dataset revealed mortality rates of 32.3% for ALI/ARDS, 24.6% for severe sepsis and 38% for those patients who presented with a primary disease complicated by acute renal failure.

## Discussion

Intensive care medicine in Ireland has been the subject of a number of publications and reports defining the nature of the service. The lack of a centralized common dataset inhibits the ability to describe this complex patient population. In contrast, in England, Wales and Northern Ireland, the Intensive Care National Audit and Research Centre, and in Scotland the Scottish Intensive Care Society Audit Group have undertaken independent audit for many years. Both of these systems have proven to be powerful tools for benchmarking and collaborative research. In the Republic of Ireland there is an urgent need for resources to support either participation in the Intensive Care National Audit and Research Centre dataset or establish an Irish system. Despite this, a number of reports have helped to define adult intensive care activity in the Republic of Ireland, specifically the Accessibilty Report [7] of 2002 and the Eastern Region Report [8] of 2004. However, all of these reports focus on the nature of service delivery rather than a description of the patient population.

The creation of intensive care facilities and resources has often been a parallel development with major elective surgery (for example, cardiac surgery and neurosurgery). However, the data in this study identify that 78% of intensive care admissions are now emergency admissions, with nearly 23% of patients admitted with sepsis. The mortality of this subgroup of patients, at 24.6%, compares favourably with international standards, particularly because 86% of patients in the sepsis subgroup have severe sepsis (sepsis plus organ dysfunction) [9]. ICU-acquired infection (new infection more than 2 days after admission) was reported in 80 (7.7%) patients. This probably represents an underestimate of the true prevalence; a more accurate estimate would require a dedicated study, with strict diagnostic criteria focused on this specific question. Recent work by Damas and coworkers [10] suggests 29% to be a more realistic figure. Our work focused on sepsis with organ dysfunction, and therefore is likely to fail to capture either infection without new organ dysfunction or new sepsis with established organ dysfunction.

The readmission rate at 7% exceeds an international benchmark [11,12] of 4%, referenced as a quality standard by the Quality Indicators in Critically Ill Patients [13] of the Spanish Society of Intensive Care. The higher rate may reflect the effect of premature discharge of patients due to pressure on ICU beds, a contention supported by the high percentage of out of hours discharges from ICU. Readmission is known to affect outcome adversely [14]. In this study, patients who were readmitted to the ICU had a mortality rate of 23%, as compared with 17.6% in patients not readmitted.

The rate of mechanical ventilation was 70.7% on admission, suggesting that the resource of intensive care is reserved for the most critically ill in Irish hospitals. This is also comparable to the ventilation rate in the Scottish Intensive Care Society Audit Group data [15] over the past 6 years. Outcomes from ALI and ARDS, with ICU mortality rates of 21% and 37.8%, respectively, are similar to those observed in major clinical trials in patients with ALI/ARDS, such the ALIVE [16] study (ICU mortality 49%) and ARDSNet [17] studies (mortality of 31% to 39.8% in a selected patient population), with a notable standardization of approach to pressure limiting of ventilation, as is the current recommended standard of practice [6].

Use of the RIFLE criteria allows an overview of the evolution of acute renal dysfunction [5,18]. It is noted that 289 (28%) patients had an AKI either on admission or during their ICU stay. Of these, 69 (6.7% of total population) required some form of RRT, with a mortality of 38% for patients within RIFLE Failure criteria. Data from the Scottish Intensive Care Society Audit Group [15] for 2005 reveal an 11% rate of RRT, and mortality was not specified. The BEST Kidney (Beginning and Ending Supportive Therapy for the Kidney) investigators, in a multicentre (54 centres) study [19] conducted across 23 countries, analyzed 1,006 patients treated with continuous RRT in intensive care. They reported a mortality rate of 32.8% on continuous RRT and a hospital mortality of 63.4% for these patients.

The rate of interhospital transfer was 8% (n = 85) for this study period, which would then approximate to greater that 450 patients per annum, not including transfers to those ICUs not participating in this dataset. A total of 72 traumatic brain injuries are described, of whom 32 were transferred to a neurosurgical centre. There appeared to be a regional variation in transfer rates, with an equivalent number of patients (n = 16) transferred to the regional neurosurgical centres in the Republic (population 4.2 million) and Northern Ireland (population 1 million). It is not possible to extrapolate from the dataset the reasons for this difference.

Limitations of the study include an inability to include all ICUs, and in relation to interhospital transfers an inability to define the selection process leading to transfer. With regard to disease definition, there was a reliance on each unit's principle investigator for reliability of data. However, all definitions were provided using the data collection tool. Of the 14 participating hospitals identified at the start of the study, four centres were unable to complete the work because of the amount of time required to complete the set for each patient on a daily basis over a 10-week period. Two of these centres were university teaching hospitals and two were smaller units. Retrospective review of their ICU admissions for the study period identifies a further 311 patient episodes not entered in the dataset, meaning that 77% of episodes were captured across the ICCTG network. Most of the ICUs do not have a data clerk or other staff member whose role is focused on data acquisition. However, we feel that a dataset of 1,029 patients across a 10-week period including 50% of all Irish ICU beds is a representative sample for describing intensive care activity for the country.

## Conclusion

This study describes, for the first time, the adult intensive care patient population across all of Ireland, North and South. The authors consider this to be an important step in achieving a collaborative research ethos across the intensive care community.

## Key messages

• Describing the national critical care population is essential to inform hypotheses, feasibility and design of multicentre clinical trials.

• The ICCTG has established a network of collaborating intensive care practices to progress multicentre clinical trials.

## Abbreviations

AKI: acute kidney injury; ALI: acute lung injury; ARDS: acute respiratory distress syndrome; CRSC: Clinical Research Support Centre; FiO_2_: fractional inspired oxygen; ICCTG: Irish Critical Care Trials Group; ICU: intensive care unit; NIV: noninvasive ventilation; PaO_2_: arterial oxygen tension; RIFLE: Risk, Injury, Failure, Loss of kidney function, End-stage kidney disease; RRT: renal replacement therapy; SOFA: Sequential Organ Failure Assessment.

## Competing interests

The authors declare that they have no competing interests.

## Authors' contributions

The study was conceived and designed by the ICCTG, as represented by the investigators listed below. All members listed acted as site lead investigators and were responsible for data collection and submission. All lead investigators and CRSC were circulated by the writing committee (B Marsh and D McAuley) and contributed to the writing of the paper. Statistical analysis was conducted by the CRSC.

The Irish Critical Care Trials Group is as follows: M Sheridan (Altnagelvin Hospital), M Donnelly (AMNCH Tallaght Hospital), R Bailie (Antrim Area Hospital), M Power (Beaumont Hospital), P Seigne (Cork University Hospital), S Austin (Mater Hospital, Belfast), B Marsh (Mater Miscericordiae University Hospital), C Motherway (Mid Western Region Hospital), M Scully (Our Lady of Lourdes Hospital), C Fagan (St James's Hospital), P Benson (St Vincent's Hospital), D McAuley (Royal Victoria Hospital), J Trinder (Ulster Hospital), J Bates (Galway University Hospitals) and K Bailie (CRSC).
